# Editorial: Personalized Medicine in Cancer Research

**DOI:** 10.3389/fendo.2018.00692

**Published:** 2018-11-20

**Authors:** Haim Werner, Teresa Wood

**Affiliations:** ^1^Department of Human Molecular Genetics and Biochemistry, Tel Aviv University, Tel Aviv, Israel; ^2^Department of Pharmacology, Physiology and Neuroscience and Cancer Center, Rutgers University, The State University of New Jersey, New Brunswick, NJ, United States

**Keywords:** personalized medicine, cancer, melanoma, human genome project, precision medicine

The aim of the *Personalized Medicine in Cancer Research* RT was to provide an overview on the trends, achievements, and challenges lying ahead in this field. Since the unveiling of the Human Genome Project we have being witnessing an extraordinary revolution in the way in which medicine is perceived, both from the patient's perspective as well as from the practitioner's standpoint. The concept of “*personalizing*” treatments holds the promise of providing the best possible avenues for disease therapy in the future.

The article entitled “*Personalized Medicine in Malignant Melanoma: Toward Patient Tailored Treatment*” by Helgadottir et al. summarizes the current knowledge on melanoma molecular classification, predictive markers, and combination therapies as well as emerging new treatments. Skin melanoma is still a major clinical challenge and, until recently, there have been limited options for effective treatment of disseminated disease. A dramatic change in the management of melanoma took place in the last decade, with significant improvements in patient outcomes and a shift toward patient-centered treatments.

Treatment of patients with gynecologic tumors diagnosed at advanced stages remains a therapeutic challenge. Survival rates remain low, despite surgery, and chemotherapy. Advances in understanding the role of the immune system in the pathogenesis of cancer have led in recent years to the evolution of immunotherapeutic approaches. The article entitled “*The Role of the Insulin-Like Growth Factor 1 Pathway in Immune Tumor Microenvironment and Its Clinical Ramifications in Gynecologic Malignancies*” by Alemi Yahya et al. provides an overview on current immunotherapy strategies and on IGF-targeted therapy for gynecologic malignancies.

Precision medicine utilizes a range of methodologies to allow real-time analysis of individual genomic signatures in primary tumors and metastases with the goal of finding clinically actionable targets. The article entitled “*Precision Medicine in Hormone Receptor-Positive Breast Cancer*” by Nasrazadani et al. discusses the opportunities and challenges in integrating precision medicine through next-generation genomic sequencing into the management of breast cancer.

Insulin-like growth factor 2 (IGF2) mRNA-binding protein 3 (IGF2BP3) is an oncofetal protein that binds RNA, thereby affecting the fate of target transcripts. Mancarella et al. investigated the effect of IGF2BP3 on the regulation of the IGF system in Ewing sarcoma. The article entitled “*Insulin-Like Growth Factor 2 mRNA-Binding Protein 3 Influences Sensitivity to Anti-IGF System Agents Through the Translational Regulation of IGF1R*” demonstrates that the detection of IGF2BP3 expression should be combined with the assessment of the IGF1R/Insulin receptor ratio to predict cell responses to anti-IGF1R agents.

Proteoglycans (PGs) are important constituents of the extracellular matrix. PGs regulate the bioavailability of hormones, growth factors, and cytokines as well as the activation of their respective receptors. PGs have been associated with cancer pathogenesis. The review entitled “*Proteoglycans-Biomarkers and Targets in Cancer Therapy*” by Nikitovic et al. discusses the roles of PGs in cancer progression, and focuses on the development of technologies aimed at defining a PG “signature” in disease. This signature may lead to the development of innovative biomarkers and selective, more efficient therapies.

Finally, the article entitled “Predictive and Prognostic Brain Metastases Assessment in Luminal Breast Cancer Patients: FN14 and GRP94 from Diagnosis to Prophylaxis” by Martínez-Aranda et al. considers the usefulness of FN14 and GRP94 expression to stratify breast cancer patients according to their risk of brain metastasis progression. Based on their data, the authors propose a new follow-up protocol for the early prevention of clinical brain metastases of breast cancer patients.

In conclusion, the *Personalized Medicine in Cancer Research* RT focuses on novel state-of-the-art technologies and their applications in cancer diagnostics and therapeutics. We trust that the information summarized here will be of help to basic scientists as well as clinicians.

## Author contributions

All authors listed have made a substantial, direct and intellectual contribution to the work, and approved it for publication.

### Conflict of interest statement

The authors declare that the research was conducted in the absence of any commercial or financial relationships that could be construed as a potential conflict of interest.

